# From Photons to Product – A Quantitative Study on Electron Fluxes in *Synechocystis* sp. PCC 6803 Applied for Oxygenase Catalysis

**DOI:** 10.1111/ppl.71126

**Published:** 2026-10-13

**Authors:** Adrian Tüllinghoff, Lieselotte Van Looveren, Jörg Toepel, Bruno Bühler

**Affiliations:** ^1^ Department of Microbial Biotechnology Helmholtz‐Centre for Environmental Research – UFZ Leipzig Germany; ^2^ Faculty I of Natural Science ‐ Biological Science Martin‐Luther‐University Halle‐Wittenberg Halle Germany

**Keywords:** cyanobacteria, electron flux, light‐driven redox biocatalysis, photosynthetic efficiency, quantitative physiology, sink effect

## Abstract

Using Cyanobacteria as chassis strains can harness sunlight as an energy source for biotechnology. Their phototrophic lifestyle requires balancing energy assimilation (source) with nutrient assimilation and biomass formation (sink). It is hypothesized that extra carbon and/or electron sinks can increase photosynthetic efficiency, allowing redox biotransformations to tap into otherwise unused photosynthetic potential. We investigated physiological responses of the cyanobacterium *Synechocystis* sp. PCC 6803 to the onset of an electron consuming biotransformation under three different sink scenarios. A heterologously expressed two‐step cascade converting cyclohexanone to 6‐hydroxyhexanoic acid served as a proxy for growth‐decoupled consumption of photosynthetically generated electrons. Specific growth rates, specific quantum absorption, and effective quantum yield at photosystem II were assessed under steady state conditions. Balancing absorbed photons and generated electrons revealed an increased alternative electron flux upon heterologous gene expression, while the biotransformation (partially) rerouted this flux toward product formation. Despite the capacity of *Synechocystis* sp. PCC 6803 to react to different sink conditions, high biotransformation rates could not be maintained, likely due to metabolic or enzymatic instabilities. Low‐carbon conditions were most suitable to distribute reductants between growth and biotransformation, whereas high‐carbon conditions enabled the highest quantum efficiencies. The physiological analysis of an oxygenation‐active cyanobacterium represents an important cornerstone to optimize phototrophic metabolism for photo‐biotechnological applications.

## 1 Introduction

Phototrophic microorganisms like cyanobacteria have been shown to be interesting hosts for biotechnological processes. Their utilization is motivated by the great promise of harnessing the sun as a power supply for production. Making use of their core metabolism, the oxygenic photosynthesis, cyanobacteria are able to form biomass based on sunlight, water, and CO_2_ as energy, electron, and carbon sources, respectively. Under environmental conditions, the availability of these resources varies. During the day, phototrophic organisms are typically exposed to light intensities above or below the light saturation point (*E*_k_), at which incident light can be used most efficiently for assimilatory processes (Wagner et al. 2006; Jakob et al. 2007; Mullineaux 2014; Wilhelm and Selmar 2011). Thus, cyanobacteria are forced to constantly balance source and sink utilization and thus energy assimilation (light harvesting, photosynthetic electron flux) and energy demand (nutrient assimilation and biomass formation). To react to sudden imbalances, cyanobacteria possess mechanisms to dissipate energy, with non‐photochemical quenching or alternative electron transfer in Mehler‐like reactions as the most prominent ones (Allahverdiyeva et al. 2015; Thiel et al. 2019). To balance source/sink ratios on the longer term, state transitions, balancing the absorbed light energy between the two photosystems, and changes in PSII‐yield, Chlorophyll‐*a* (Chl‐*a*) content, and Rubisco content are observed (Mullineaux 2014; Jakob et al. 2007; Wilhelm and Selmar 2011). On the one hand, these balancing processes and dissipation mechanisms protect the cell from photo‐inhibition, that is, light‐induced damage of the photosynthetic apparatus. On the other hand, they encompass an energy loss, reducing the overall light conversion efficiency of phototrophs (Barber 2009), with photosynthesis operating below its true potential.

To increase photosynthetic efficiency, sink engineering has recently become popular. It is based on the sink hypothesis stating that photosynthetic efficiency can be increased by providing productive pathways as energy/electron sinks making use of surplus photosynthetic energy. In principle, two approaches are pursued: (i) **CO**_**2**_ **‐based production** of organic chemicals for material and energy use (Liu et al. 2022; Oliver and Atsumi 2015; Savakis et al. 2015; Ducat et al. 2012; Abramson et al. 2016; Grund et al. 2022) and (ii) **redox biotransformations** directly tapping photosynthetic electron supply (Hoschek et al. 2019; Assil‐Companioni et al. 2020; Berepiki et al. 2016; Tüllinghoff et al. 2022; Toepel et al. 2023).

CO_2_‐based production makes use of both the photosynthetic light reaction and the Calvin‐Benson‐Bassham (CBB) cycle to convert CO_2_ to products such as butanol (Liu et al. 2022), coumaric acid (Kukil et al. 2023), sucrose (Lin et al. 2020), isoprene (Janssen et al. 2025), or lactate (Grund et al. 2022), as reviewed by Tan et al. (2022). Utilizing the greenhouse gas CO_2_ as carbon source, this strategy sets a basis for highly sustainable processes.

Interestingly, studies on heterologous pathways as carbon sinks in cyanobacteria describe enhanced carbon fixation compared to the respective wild‐type strains (Ducat et al. 2012; Oliver and Atsumi 2015; Grund et al. 2022), clearly indicating that cells do not seize their full metabolic potential during ordinary growth and that carbon sink engineering can increase photosynthesis efficiency. In a previous study, it was shown that, under high‐light/low‐carbon conditions reflecting quasi natural conditions, heterologous lactate synthesis in the cyanobacterium *Synechocystis* sp. PCC 6803 (*Synechocystis*) increased carbon assimilation without affecting biomass formation (Grund et al. 2022). To meet the increased sink demand, PSII‐yield and photosynthesis‐derived electron flux were found to be enhanced by 24% and 28%, respectively (Grund et al. 2022). CO_2_‐based production is however tightly linked to CO_2_ uptake and fixation capacities, limiting production rates for carbon‐rich products to values typically below 20 mg g_CDW_^−1^ h^−1^ (CDW, cell dry weight) and depending on CO_2_ enrichment (Angermayr et al. 2015).

As an approach independent from CO_2_ fixation and thus enabling higher production rates, the utilization of photosynthesis‐derived electrons has gained increasing attention in the last years (Jodlbauer et al. 2021; Hoschek et al. 2019; Assil‐Companioni et al. 2020; Berepiki et al. 2016). Thereby, oxidoreductase‐based biotransformations are linked to the light reaction and create a reductant sink that is decoupled from assimilatory routes like the CBB cycle (Theodosiou et al. 2022). Maximum specific production rates range from 240–420 mg g_CDW_^−1^ h^−1^ using cytochrome P450 (Hoschek et al. 2019) and Baeyer‐Villiger monooxygenases (Tüllinghoff et al. 2022) to 1000 mg g_CDW_^−1^ h^−1^ using ene‐reductases (Assil‐Companioni et al. 2020), which reduce carbon–carbon double bonds. These numbers illustrate the high availability of photosynthesis‐derived electrons for heterologous redox reactions, recently being estimated to withdraw up to 50% of electrons released from water splitting (Hubacek et al. 2024). However, reported production periods range from minutes to a few hours. On the longer term, high electron supply rates could not be maintained, reducing specific activities feasible in the long term (> 24 h) to 10–25 U g _CDW_^−1^ (corresponding to 60–150 mg g_CDW_^−1^ h^−1^ for ~100 g mol^−1^ products; Hoschek et al. 2019; Tüllinghoff et al. 2022; Tüllinghoff et al. 2023). Both light limitation (Hoschek et al. 2019; Hobisch et al. 2021) and reactant toxicity (Hoschek et al. 2019; Tüllinghoff et al. 2022) were reported as factors affecting photosynthetic electron supply in the long run.

Intriguingly, activating extra electron sinks led to an increase of photosynthetic rates, reflected by enhanced PSII‐yield (Berepiki et al. 2016; Grund et al. 2019; Santos‐Merino et al. 2021), quantum absorption (Grund et al. 2019), and photosynthesis‐derived electron flux (Berepiki et al. 2016; Grund et al. 2019; Santos‐Merino et al. 2021). Like carbon sinks, mere electron sinks can evidently relieve the sink limitation and increase photosynthetic efficiency. In a previous study, examining physiological differences upon nitrogen assimilation from NO_3_^−^ and NH_4_^+^, a strong electron demand posed by NO_3_^−^ assimilation was found to involve higher photosynthetic rates, which also promoted carbon fixation and growth, indicating a favorable sink/source balance (Grund et al. 2019). Accordingly, an electron‐consuming biotransformation may benefit the photosynthetic metabolism by utilizing electrons derived from water that become available as consequence to upregulated photosynthetic activity. To test this, we used an orthogonal two‐step cascade for the conversion of cyclohexanone (C‐one) via ε‐caprolactone (ε‐Cl) to 6‐hydroxyhexanoic acid (6‐HA) by means of recombinant Baeyer‐Villiger monooxygenase (BVMO) and lactonase in *Synechocystis* (Tüllinghoff et al. 2023). This cascade has been shown to increase energy conversion efficiencies when the biotransformation substrate was provided as an extra electron sink. Interestingly, the rate of electron withdrawal was found to be decisive for metabolic and bioconversion stability: A moderate electron withdrawal rate was tolerated over an extended period of 48 h, while a higher electron withdrawal rate posed a too strong metabolic burden (Tüllinghoff et al. 2023).

In the context of photo‐biotechnology for light‐driven production of both high volume chemicals like polymer precursors and energy carriers like H_2_ (Kannchen et al. 2020), it is of fundamental interest, how electrons are partitioned between growth metabolism and heterologous biotransformation and which conditions enable sustained electron withdrawal over a longer time period, that is, several days or weeks. To answer this question, metabolic prerequisites to stabilize electron withdrawal over extended time periods and factors influencing the metabolic robustness have to be investigated.

In this study, we investigated the above‐described two‐step cascade in *Synechocystis* as a proxy for an electron sink linked to the photosynthetic light reaction. To get insight into the electron flux distribution in catalytically active cells and to determine to what extent electrons can be decoupled from cyanobacterial growth, different steady states were established during continuous cultivation assessing cell physiology in terms of growth rate, O_2_ formation, carbon fixation, PSII‐yield, and quantum absorbance. Initially, the available photosynthetic capacity was increased via light irradiance elevation at constant sink availability (without biotransformation), reflected by energy tap modulation in Figure 1. In a second step, cells were exposed to stable source (light intensity) conditions altering sink demands for C‐fixation, N‐assimilation, and biotransformation. This allowed identifying a tradeoff regarding source–sink balancing: While high‐carbon (sink‐replete) conditions enable high short‐term electron flux toward the biotransformation, low‐carbon (sink‐limited) conditions provide greater long‐term stability, albeit at lower conversion rates.

**FIGURE 1:**
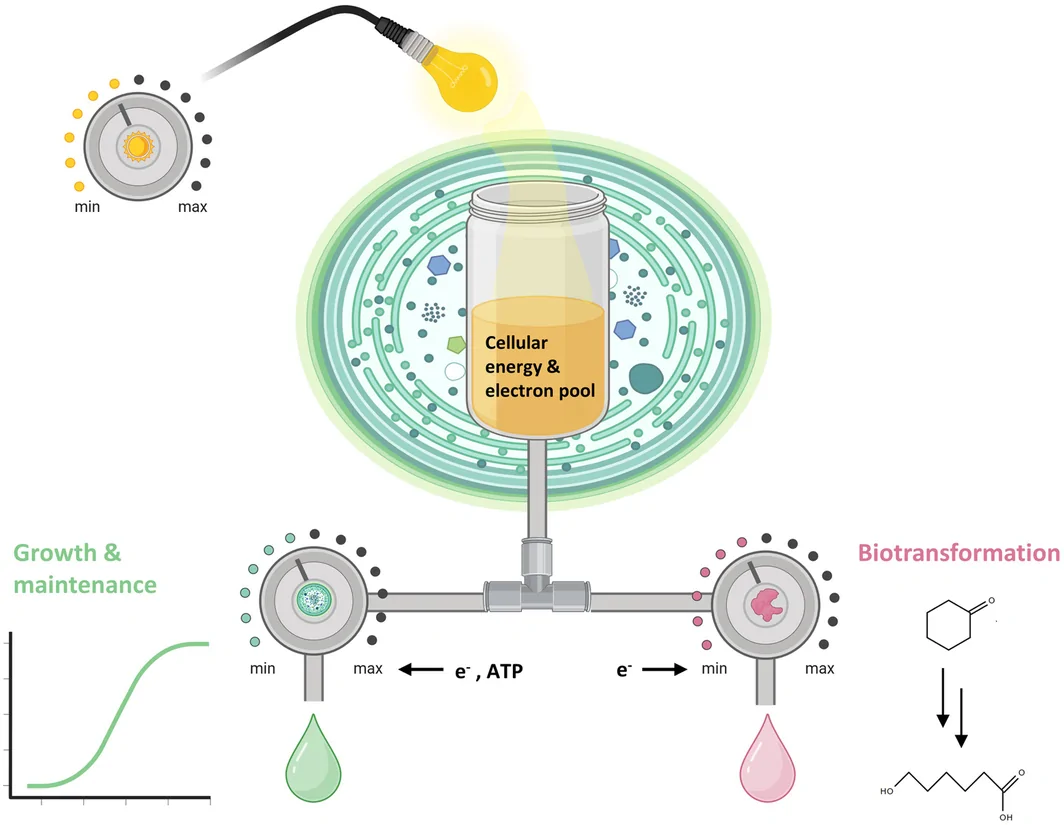
Source/sink model of a phototrophic oxygenation‐active biocatalyst. The container allegorizes the electron/energy (e^−^/ATP) pool which is filled by light energy (source) tapped by photosynthesis. The resulting pool can be used for growth and maintenance and/or for a redox‐biotransformation representing the sinks. Naturally, light source and the “growth & maintenance” sink have to be balanced under varying source/sink conditions, that is, light intensity and sink availability, especially CO_2_ and N‐sources. The applied biotransformation constitutes an additional electron sink, which possibly can exploit unused photosynthetic potential according to the sink hypothesis (Figure created in BioRender).

## 2 Experimental Section

### 2.1 Bacterial Strains

A previously designed *Synechocystis* strain co‐expressing Baeyer‐Villiger monooxygenase and lactonase genes from *Acidovorax* sp. CHX100 (Tüllinghoff et al. 2023), *Synechocystis* BVMO:Lactonase, and the corresponding wild‐type (*Synechocystis* wt) were maintained as cryo‐cultures at −80°C in BG11 medium with 20% dimethyl sulfoxide and were freshly cultivated prior to the experiments (Shcolnick et al. 2007).

### 2.2 Growth Conditions

*Synechocystis* strains were cultivated as previously described (Tüllinghoff et al. 2023). BG 11 medium supplemented with 10 mM or 5 mM 4‐(2‐hydroxyethyl)‐1‐piperazinethanesulfonic acid (HEPES), pH 7.5, was used for shaking flask or bioreactor cultivations, respectively. To enable correct carbon balancing, Na_2_CO_3_ was omitted. For all experiments using NH_4_^+^ as an N source, NaNO_3_ was substituted with NH_4_Cl (final concentration 17.5 mM). Standard cultivation conditions were 50 μmol photons m^−2^ s^−1^, 30°C, 75% humidity, and ambient CO_2_ levels (0.04% [v/v]) for shaking flask experiments and bioreactor precultures (Multitron; Infors AG).

Continuous cultivations were conducted in flat‐panel (FP) bioreactors (Labfors 5 Lux; Infors AG, light path length 2 cm) at a working volume of 1.8 L as described before (Grund et al. 2022) with altered standard conditions: 30°C, pH 7.5 (controlled with 1 M HCl and 1 M NaOH), and aeration at 900 mL min^−1^ (0.5 vvm). To reduce the O_2_ share in the aeration gas for precise O_2_ evolution measurements, 860 mL min^−1^ N_2_ and 40 mL min^−1^ pressurized air were premixed in a gas mixing station (GMS150; Photon System Instruments) and fed to the reactor. As a second aeration gas, 10 mL min^−1^ of pure CO_2_ or 10% CO_2_/90% N_2_ for high and low carbon conditions were provided, resulting in 0.6 and 0.12% CO_2_ (v/v), respectively. Bioreactors were illuminated by white light LEDs allowing stepless controlling of the incident light intensity. To keep the input light energy constant, a turbidostat mode was conducted by feeding fresh medium via a peristaltic pump into the reactor at a rate equaling the specific growth rate under respective conditions. Another option for continuous cultivation would be a chemostat, in which a limitation in a media component (such as Mg, K, P) keeps biomass concentrations constant. Under these conditions, however, the light input would not necessarily have been constant, so they were not used in the study.

The reactor filling level was kept constant by removing culture broth through a fixed efflux tube at the top of the cultivation vessel. The cell concentration was kept below 200 mg_CDW_ l^−1^ to minimize self‐shading and thus ensure even light supply. For every sink scenario, a new turbidostat bioreactor was started by inoculation from a batch pre‐culture. Biotransformation was initiated by starting a separate substrate feed of 50 mM C‐one in the respective BG11 medium, synchronized with the medium feed. Feed rate, pH, and pO_2_ were continuously monitored online using EVE software. The following parameters were measured on three different sampling days for each steady state in every sink scenario or at specific time points for all biotransformation phases.

### 2.3 Biomass, Chl‐*a*, and Carotenoid Concentrations

Biomass concentrations were assessed by cell dry weight (CDW), OD_750_, and cell count measurements (Multisizer 4E, Beckman Coulter) as previously described (Grund et al. 2019; Tüllinghoff et al. 2022). The growth rate in continuous mode was determined from the dilution rate D and the changes in biomass concentration (dX) according to (Equation 1) with X_1_ and X_2_ being biomass concentrations determined at time points 1 and 2, respectively:

(1) $$\mu_{1,2}=\frac{\mathrm{dX}}{\mathrm{dt}}\frac{1}{\frac{X_{1}+X_{2}}{2}}+D$$

A new steady state was considered to have been reached when the growth rate μ was constant (less than 5% deviation) for at least three culture volume exchanges. Extractions of Chl‐*a* and carotenoids were conducted as described (Grund et al. 2019) and concentrations were calculated according to Zavřel et al. (2015).

### 2.4 Carbon Fixation and Oxygen Evolution Rates

Carbon fixation and oxygen evolution rates were determined by measuring O_2_ and CO_2_ concentrations in the in and off gas with BlueInOne sensors (BlueSens). Additionally, O_2_ concentrations were measured using optical O_2_ sensors with temperature corrections (FireSting; PyroScience).

### 2.5 Substrate and Product Concentrations

C‐one, C‐ol, ε‐Cl, and 6‐HA concentrations were determined as described previously (Tüllinghoff et al. 2022). For C‐one, C‐ol, and ε‐Cl quantification, samples taken at specific time points were quenched and extracted by adding one volume of diethylether containing 0.2 mM n‐decane as internal standard, followed by drying over Na_2_SO_4_, transfer to gas chromatography (GC) vials, and GC measurements according to the procedure of Tüllinghoff et al. (2022). For 6‐HA quantification, samples were quenched by adding 0.11 volumes of acetonitrile and centrifuged for 1 min at 13,000 × g. Supernatants were transferred to high‐pressure liquid chromatography (HPLC) vials for HPLC measurements.

To calculate product formation rates (including specific activities) during continuous cultivation, apparent linear product accumulation in a specific time range was considered by calculating an average outflow product concentration (C_out_) via (Equation 2):

(2) $$C_{\text{out}}=\frac{c_{1}+c_{2}}{2}D\;\left(t_{2}-t_{1}\right)$$

with D being the dilution rate [h^−1^], and c_1_ and c_2_ the product concentrations [mM] at time points t_1_ and t_2_ [h]. Specific activities in this time range were then determined by dividing the product outflow rate based on the outflow concentration ($C_{\text{out}}*D$) by the steady state biomass concentration. 1 U equals the formation of 1 μmol_6‐HA_ min^−1^.

### 2.6 Effective Quantum Yield of PS II and Quantum Absorption

The effective quantum yield at PSII (Φ_PSII_) was measured and calculated as previously described using a Multi‐Color Pulse amplitude modulation (MC‐PAM) device (Heinz Walz GmbH; Grund et al. 2019). Chl‐*a* fluorescence was measured via excitation at 400 nm in order to minimize excitation of phycobilisomes at higher wavelengths and thus underestimation of Φ_PSII_ (Ogawa et al. 2017; Schuurmans et al. 2014). Samples from the bioreactor were transferred instantly into the measuring cuvette of the MC‐PAM to determine Φ_PSII_ in the light‐acclimated state of the cells. For Φ_PSII_ assessment, Chl‐*a* fluorescence was then continuously measured as a light induction curve, during which white actinic light was step‐wise increased every 30 s from 2 to 20, 80, 114, 156, 220, and 290 μmol_photons_ m^−2^ s^−1^. Before each actinic light step, a saturation pulse of 300 ms was applied. White actinic light was used to provide conditions in the measurement cuvette that are comparable to bioreactor conditions.

Quantum absorption was determined from the difference between incident and transmitted light. Transmitted light was measured using a quantum sensor (LI‐COR Biosciences) positioned behind the reactor, and sensor output was continuously recorded via the reactor control unit. Time‐averaged values, corrected for abiotic light absorption, were used to calculate the specific total quantum absorption (Q_phar_) at each sampling time based on the corresponding biomass concentration.

According to the Beer–Lambert law (Equation 3), the light intensity is attenuated when passing through the reactor depending on the distance d and the cell concentration c:

(3) $$I\left(d\right)=I_{0}\;e^{-\varepsilon *c*d}\;\left[{\text{μmol}}_{\text{photons}}\;m^{-2}\;s^{-1}\right]$$

This has to be considered, as determining Φ_PSII_ on the basis of the incident light intensity I_0_ leads to underestimation of Φ_PSII_. While the extinction coefficient ε is unknown, the product ε * c remains constant in a steady state during continuous cultivation. Thus, the light scattering/absorption by the cell suspension could be quantified at known I_0_ and transmission values recorded by the quantum sensor at *d* = 2 cm constituting the horizontal diameter of the reactor. To correct Φ_PSII_, the available light depending on the horizontal position d [in cm] in the cultivation chamber was assessed and used to derive differential data for Φ_PSII_. These differential data were then averaged according to (Equation 4).

(4) $$\phi_{\text{PSII},\;\text{corr}}=\bar{\sum_{d=0\mathrm{cm}}^{2\mathrm{cm}}\phi_{\text{PSII}}\left(I\left(d\right)\right)}$$

### 2.7 Balancing Electron Fluxes in Cyanobacteria

The maximum electron flux through PS II r_F_ was calculated from Φ_PSII,corr_ determined by PAM‐fluorometry, and Q_phar_ via (Equation 5):

(5) $$r_{F}=\Phi_{\text{PSII},\;\text{corr}}*\frac{Q_{\text{phar}}}{2}\;\left[{\text{mmol}}_{e-}\;{g_{\mathrm{CDW}}}^{-1}\;h^{-1}\right]$$

considering the fact that (at least) two quanta have to be absorbed per electron, assuming linear electron transfer. The electron flux to carbon and nitrogen assimilation, defined as r_C_ and r_N_, was calculated using Equations (6) and (7):

(6) $$r_{C}=\mu *\frac{x_{C}}{M_{C}}*4\;\left[{\text{mmol}}_{e-}\;{g_{\mathrm{CDW}}}^{-1}\;h^{-1}\right]$$

(7) $$r_{N}=\mu *\frac{x_{N}}{M_{N}}*8\;\left[{\text{mmol}}_{e-}\;{g_{\mathrm{CDW}}}^{-1}\;h^{-1}\right]$$

with the specific growth rate μ, the C and N mass fractions x_C_ and x_N_ in biomass, respectively, and the molar masses of C and N as M_C_ and M_N_, respectively. The factors 4 and 8 account for the electron demand per assimilated C‐ and N‐atom from CO_2_ and NO_3_, respectively.

Based on the product formation rate p, the electron flux to the biotransformation r_BT_ was quantified with (Equation 8):

(8) $$r_{\mathrm{BT}}=p*2\;\left[{\text{mmol}}_{e-}\;{g_{\mathrm{CDW}}}^{-1}\;h^{-1}\right]$$

with 2 mol electrons being required to form 1 mol product.

The difference between the maximum electron flux through PS II and the different sink‐related electron fluxes (r_C_, r_N_, r_BT_) is defined as non‐sink‐related electron flux r_NEF_ according to (Equation 9):

(9) $$r_{\mathrm{NEF}}=r_{F}-r_{C}-r_{N}-r_{\mathrm{BT}}\;\left[{\text{mmol}}_{e-}\;{g_{\mathrm{CDW}}}^{-1}\;h^{-1}\right]$$

It subsumes all alternative electron fluxes, such as the pseudo‐cyclic electron flux, which transfers electrons back to O_2_, as well as negligible electron sinks, such as S and P assimilation.

## 3 Results

### 3.1 Dependence of Growth Rate on Light Intensity Under Steady‐State Conditions

In order to find the optimal light intensity for the applied setup, the light saturation point (*E*_k_) of growing *Synechocystis* was determined in an initial experiment. *E*_k_ is defined as the light intensity just sufficient to supply the metabolism with required reductants (Ritchie 2008). Consequently, *E*_k_ can be seen as the optimal light intensity, as it marks the point enabling the exploitation of the full biological capacity without unnecessary energy losses via dissipation mechanisms. Light penetration and accessibility, but also the amount and type of electron sinks, depend on the chosen setup and conditions and make *E*_k_ system‐dependent.

To determine *E*_k_ for our setup, light intensities of 50, 100, 150, 250, and 500 μmol_photons_ m^−2^ s^−1^ were applied during continuous cultivation under high CO_2_ (HC) conditions (0.6% (v/v) in IN‐gas), keeping cell density, aeration, temperature, volume, and pH constant. Between 50 and 250 μmol_photons_ m^−2^ s^−1^, the growth rate μ strongly depends on the incident light intensity (Figure S1), constituting the linear branch of the μ/I‐curve. Higher light intensities of 500 μmol_photons_ m^−2^ s^−1^ did not increase μ significantly, indicating light saturation and possibly photo‐inhibition. These data suggest E_k_ being at or just above 250 μmol_photons_ m^−2^ s^−1^, where the linear branch transitions into light saturation due to energy dissipation. Notably, high light intensities (> 200 μmol_photons_ m^−2^ s^−1^) induced rapid biofilm formation, complicating an accurate assessment of active biomass and thus a correct determination of physiological rates. Therefore, we decided to apply 150 μmol_photons_ m^−2^ s^−1^ as a light intensity below *E*_k_ under standard conditions (high CO_2_, NO_3_^−^ as N‐source, no biotransformation), enabling high photosynthetic efficiency in a source‐limited regime.

### 3.2 Competition for Reductants Under High‐Carbon (HC) Conditions With NO_3_ ^−^ as N Source

The first sink scenario tested was based on CO_2_ in excess (0.6% (v/v) IN‐gas) and NO_3_^−^ as N‐source. These conditions (i) ensured that CO_2_ did not limit growth, (ii) imposed a high electron demand for CO_2_ and NO_3_^−^ reduction (NO_3_^−^ requires 8 mol e^−^ per mol assimilated N), and (iii) thus constitute light‐limited conditions. Our previous study showed that *Synechocystis* BVMO:Lactonase can increase photosynthetic rates to meet the increased electron demand for NO_3_^−^ instead of NH_4_^+^ assimilation, even under light‐limited conditions (Grund et al. 2019). In this sink scenario, *Synechocystis* BVMO:Lactonase showed fast growth (*μ* = 0.088 ± 0.003 h^−1^, Figure 2A), high quantum absorption (*Q*_phar_ = 58.2 ± 1.0 mmol e^−^ g_CDW_^−1^ h^−1^, Figure 2D), high PSII‐yield (Φ_PSII_ = 0.536 ± 0.025, Figure 2G), and thus a high rate of photosynthetic electron supply. Upon Ni^2+^‐dependent induction of heterologous BVMO and lactonase gene expression, μ slightly decreased by 6% to 0.073 ± 0.008 h^−1^. Concomitantly, Q_phar_ (55.2 ± 2.5 mmol e^−^ g_CDW_^−1^ h^−1^) and Φ_PSII_ (0.524 ± 0.055) decreased by 5% and 2%, respectively (Table 1).

**FIGURE 2:**
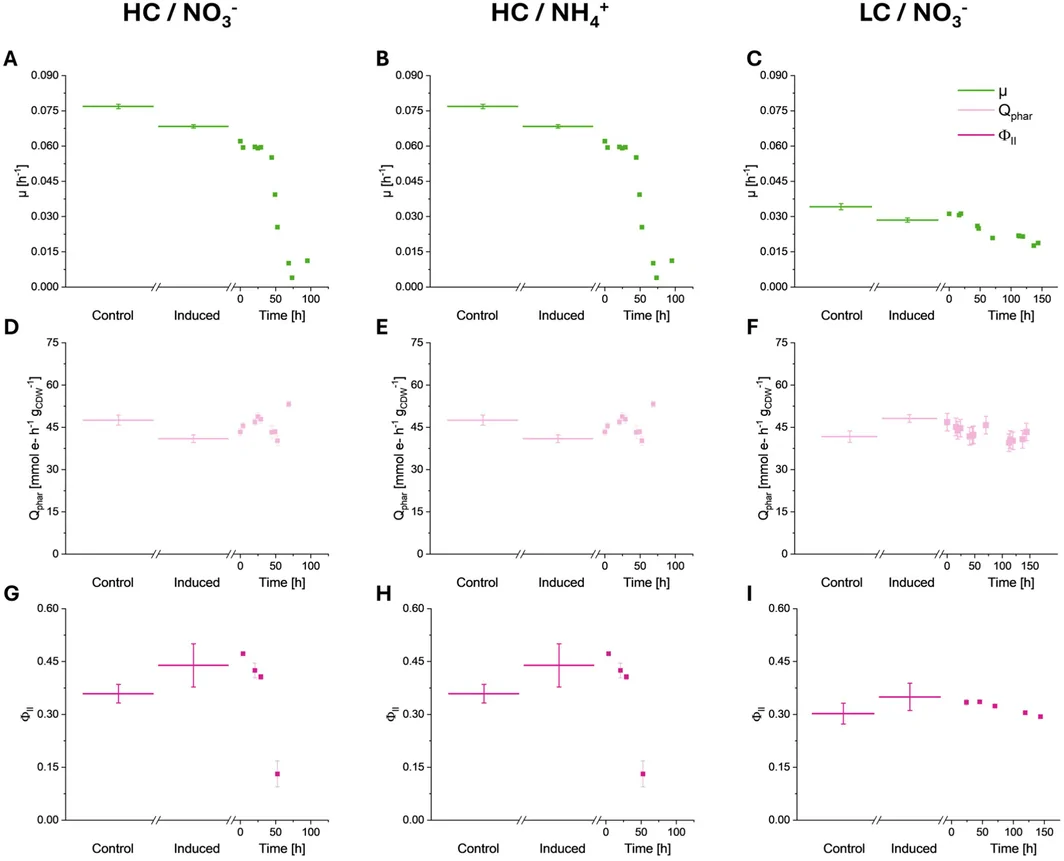
Growth rate (μ, A–C), specific quantum absorption (Q_phar_, D–F), and PSII‐yield (Φ_II_, G–I) in different sink scenarios. Cultures were grown in flat‐panel bioreactors under steady state conditions as described in the experimental section, and different conditions were applied: Scenario I (A, D, G): High carbon (HC)/NO_3_^−^, scenario II (B, E, H): HC/NH_4_^+^, scenario III (C, F, I): Low carbon (LC)/NO_3_^−^. For non‐induced (“Control”) and induced (“Induced”) conditions, steady states were established, for which means and standard deviations from three different measuring days are given. From the start of substrate feeding, time‐specific data points with standard deviations from ≥ 2 technical replicas are given with respect to a time scale [in h] on the *x*‐axis.

**TABLE 1:** Physiological characteristics of *Synechocystis* BVMO:Lactonase and *Synechocystis* wt under different source/sink conditions.

Parameter^a^	HC/NO_3_^−^ ^b^			HC/NH_4_^+^ ^b^			LC/NO_3_^−^ ^b^			Wt (HC/NO_3_^−^)^b^	
	Non‐induced	Induced	Biotrafo, mid	Non‐induced	Induced	Biotrafo, mid	Non‐induced	Induced	Biotrafo, mid	−Ni^2+^	+Ni^2+^
D = μ [h^−1^]	0.088 ± 0.003	0.073 ± 0.008	0.056 ± 0.05	0.077 ± 0.004	0.064 ± 0.004	0.048 ± 0.008	0.034 ± 0.002	0.029 ± 0.001	0.026 ± 0.001	0.103 ± 0.006	0.102 ± 0.005
X [mg_CDW_ L^−1^]	164 ± 7	179 ± 1	158 ± 6	158 ± 2	195 ± 2	167 ± 8	183 ± 16	201 ± 3	223 ± 14	166 ± 2	164 ± 5
c_Chl‐*a*_ [mg_Chl‐*a*_ L^−1^]	2.35 ± 0.05	2.52 ± 0.06	—	1.78 ± 0.11	1.94 ± 0.03	1.25 ± 0.35	1.77 ± 0.07	2.22 ± 0.13	1.58 ± 0.08	2.51 ± 0.06	2.59 ± 0.15
Chl‐*a*_CDW_ [μg_Chl‐*a*_ mg_CDW_^−1^]	22.0 ± 2.8	23.5 ± 1.8	—	14.5 ± 1.7	16.9 ± 0.2	10.3 ± 0.6	14.2 ± 0.7	14.2 ± 1.3	10.5 ± 0.7	15.4 ± 0.7	15.9 ± 0.9
Car_CDW_ [μg_Car_ mg_CDW_^−1^]	10.7 ± 1.3	12.1 ± 0.9	—	5.31 ± 0.60	8.64 ± 0.04	4.74 ± 0.19	12.4 ± 0.6	10.2 ± 0.9	11.6 ± 0.8	5.45 ± 0.24	5.33 ± 0.30
Q_*phar*_ [mmol_e‐_ g_CDW_^−1^ h^−1^]	58.2 ± 1.0	55.2 ± 2.5	55.4 ± 2.6	47.6 ± 2.8	41.0 ± 2.1	42.3 ± 1.5	41.7 ± 2.3	48.1 ± 1.4	40.0 ± 3.3	48.9 ± 4.0	52.2 ± 3.7
Φ_PSII_	0.54 ± 0.03	0.53 ± 0.06	0.30 ± 0.05	0.36 ± 0.03	0.44 ± 0.06	0.24 ± 0.09	0.30 ± 0.04	0.35 ± 0.04	0.34 ± 0.01	0.56 ± 0.01	0.57 ± 0.02
r_F_ [mmol g_CDW_^−1^ h^−1^]	31.2 ± 3.1	29.1 ± 4.4	16.8 ± 2.5	17.1 ± 2.1	18.0 ± 2.5	10.2 ± 5.4	12.6 ± 2.1	16.8 ± 2.3	13.4 ± 1.5	27.4 ± 2.8	29.6 ± 3.0
r_C_ [mmol g_CDW_^−1^ h^−1^]	15.0 ± 0.8	12.3 ± 0.6	9.5 ± 0.8	13.1 ± 0.7	11.6 ± 0.6	8.2 ± 0.4	5.8 ± 0.3	4.9 ± 0.3	4.4 ± 0.2	17.5 ± 0.9	17.4 ± 0.9
r_N_ [mmol g_CDW_^−1^ h^−1^]	5.7 ± 0.3	4.7 ± 0.3	3.6 ± 0.2	—	—	—	2.5 ± 0.1	2.1 ± 0.1	1.9 ± 0.1	6.7 ± 0.4	6.6 ± 0.3
r_NEF_ [mmol g_CDW_^−1^ h^−1^]	10.6 ± 1.7	12.1 ± 4.4	1.8 ± 1.1	4.0 ± 1.9	6.4 ± 3.1	0.9 ± 5.7	4.3 ± 1.9	9.2 ± 2.1	7.2 ± 1.3	3.3 ± 2.8	5.6 ± 3.0
r_BT_ [mmol g_CDW_^−1^ h^−1^]	—	—	1.8 ± 0.2	—	—	1.0 ± 0.1	—	—	0.5 ± 0.1	—	—
QE_6‐HA_ [%]	—	—	2.1 ± 0.3	—	—	1.5 ± 0.3	—	—	0.8 ± 0.2	—	—
Q_C_ [mol_quanta_ mol_C_^−1^]	31.0 ± 2.2	35.9 ± 3.4	46.7 ± 6.1	29.1 ± 3.3	28.3 ± 2.9	41.3 ± 7.5	57.5 ± 6.1	80.2 ± 7.3	72.6 ± 7.6	22.4 ± 3.0	24.0 ± 2.9

^a^ D, dilution rate equaling the specific growth rate μ; X, biomass concentration; c_Chl‐*a*_, volumetric Chl‐*a* concentration; Chl‐*a*_CDW_ and Car_CDW_, biomass‐specific Chl‐*a* and carotenoid concentrations, respectively; Q_phar_, quantum absorption rate expressed as linear electron transfer rate (2 photons ≡ 1 e^−^); Φ_PSII_, effective quantum yield at PSII; r_F_, fluorescence‐based electron flux at PSII; r_C_ and r_N_, electron fluxes for carbon and nitrate assimilation, respectively; r_NEF_, non‐sink‐related electron flux; r_BT_, electron flux into product formation; QE_6‐HA_, quantum efficiency of product formation; Q_C_, quantum requirement for carbon fixation.

^b^ The tested conditions refer to high carbon and nitrate (HC/NO_3_^−^), high carbon and ammonium (HC/NH_4_^+^), and low carbon and nitrate (LC/NO_3_^−^). The wild type was tested under HC/NO_3_^−^ conditions. Data are depicted as mean values and standard deviations from ≥ 3 technical replica. See experimental section for details.

Then, the effect of amending the biotransformation was investigated by supplying C‐one in a substrate‐limited manner at a rate corresponding to an activity of 15 U g_CDW_^−1^ and thereby to an additional e^−^‐demand accounting for approximately 13% of the electron flux toward carbon fixation (r_C_ upon induction, Table 1). The *Synechocystis* strain used expresses a heterologous two‐step enzyme cascade, consisting of a BVMO and a lactonase from *Acidovorax* sp. CHX100, and is capable of converting C‐one to 6‐HA without accumulating the intermediate ε‐Cl (Tüllinghoff et al. 2023). Consequently, as expected, no ε‐Cl accumulation was detected in any of the experiments in this study. The first conversion step requires 1 mol of NADPH (i.e., 2e^−^) per mol of C‐one converted. Further, formation of the inhibitory side‐product C‐ol is prevented by operating the biotransformation in a substrate‐limited mode.

Within the first 48 h of the biotransformation, Q_phar_ remained high under these high carbon HC/NO_3_^−^ conditions (53–59 mmol e^−^ g_CDW_^−1^ h^−1^, Figure 2D), indicating constant and high reductant supply. However, because biotransformation and nutrient assimilation compete for this reductant, a decreased μ of 0.066 ± 0.001 h^−1^ was recorded within the first 24 h of biotransformation, followed by an abrupt decline to less than 0.04 h^−1^ (Figure 2A). At the same time, the electron demand of the biotransformation could no longer be met, leading to a reduced specific activity, experimentally met by a reduced substrate feed. Finally, substrate accumulated even at the reduced substrate feed (Figure 3A), which—concomitantly with still decreasing growth rates—toxified the cells, making continuous cultivation no longer possible. During biotransformation, Φ_PSII_ values were lower compared to the induced reference, but did recover as soon as the biotransformation burden (i.e., the substrate feed) was reduced at *t* = 73 h (Figure 2G). In conclusion, the combined high electron demands for N‐ and C‐assimilation and biotransformation could not be sustained by the photo‐biocatalyst in the long term under the applied light‐ and thus source‐limited conditions.

**FIGURE 3:**
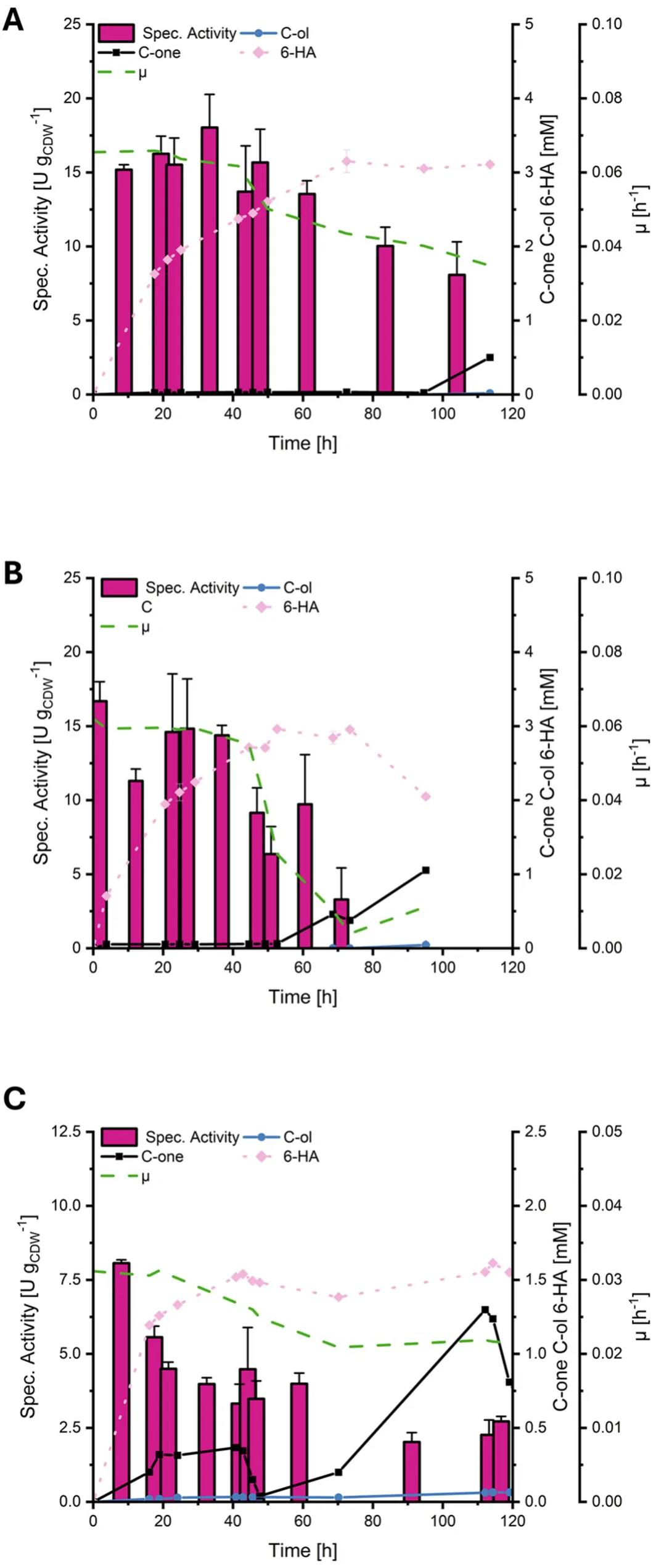
Bioconversion of C‐one to 6‐HA during continuous cultivation under (A) HC/NO_3_^−^, (B) HC/NH_4_^+^, and (C) LC/NO_3_^−^ conditions. Cultures were grown in flat panel bioreactors under steady state conditions as described in the experimental section. After recording reference and induced steady states, biotransformation was started at t = 0 h by continuously feeding C‐one into the reactor in a substrate‐limited manner at a rate matching a specific activity of 15 U g_CDW_^−1^ for HC/NO_3_^−^ and HC/NH_4_^+^ conditions and, for HC/NO_3_^−^ conditions, a rate matching 8.4 U g_CDW_^−1^, which was reduced to 4.6 U g_CDW_^−1^ at *t* = 19 h (black arrow). For every condition, a new reactor was started. Concentrations [mM] are given as solid black line/black squares for C‐one, dotted light pink line/diamonds for 6‐HA, and solid blue lines/circles for cyclohexanol (C‐ol). Dashed green lines and magenta bars represent μ [h^−1^] and specific activities [U g_CDW_^−1^], respectively. Means and standard deviations from ≥ 2 technical replica are given. Mind the different y‐axis dimensions in panel C. HC—high carbon, LC—low carbon.

### 3.3 Reducing N Assimilation Costs via NH_4_ ^+^ Provision Causes Physiological Stress Responses Upon Ni^2+^‐Based Induction of Recombinant Gene Expression

In a second scenario, the electron demand for cell growth was reduced by using NH_4_^+^ instead of NO_3_^−^ as the nitrogen source, while supplying CO_2_ in excess (0.6% (v/v) in IN‐gas). The electron demand for growth thereby decreased by 20%–25%, theoretically excelling the demand for the biotransformation. While NH_4_^+^ is known to potentially affect cell metabolism (Drath et al. 2008), similar growth rates have been observed with NO_3_^−^ and NH_4_^+^ tested as N sources under various source/sink conditions (Grund et al. 2019).

In this scenario, *μ* (0.077 ± 0.004 h^−1^, Figure 2B), *Q*_phar_ (47.6 ± 2.8 mmol e^−^ g_CDW_^−1^ h^−1^, Figure 2E), and Φ_PSII_ (0.364 ± 0.028, Figure 2H) were lower compared to the provision of the e^−^‐intensive N source NO_3_^−^, as previously observed (Grund et al. 2019). Interestingly, the growth rate decreased by 20% (to 0.064 ± 0.004 h^−1^, Table 1) upon induction of heterologous gene expression, which was more pronounced than with NO_3_^−^ (6%). Reduction of *Q*_phar_ by 14% (to 41.0 ± 2.1 mmol e^−^ g_CDW_^−1^ h^−1^) appeared to be counteracted by a higher Φ_PSII_ (0.439 ± 0.061, +21%) and was connected with a high non‐sink‐related electron flux (r_NEF_), indicating an increased energy demand. Notably, Ni^2+^ addition in combination with NH_4_^+^ as N source reduced the specific Chl‐*a* content (Table 1), as suggested by absorption spectrum shifts (Figure S2), indicating detrimental effects of Ni^2+^ and/or induction in combination with NH_4_^+^ as N source.

As in scenario I, the electron demanding biotransformation was initiated by substrate supply matching a specific activity of 15 U g_CDW_^−1^ (corresponding to 16.5% of the r_C_ upon induction, Table 1). Compared to the induced reference, the growth rate slightly decreased (by 3%–8%) to 0.059–0.062 h^−1^ within the first 48 h (Figure 2B). In the same time range, *Q*_phar_ increased up to 48.8 ± 4.1 mmol e^−^ g_CDW_^−1^ h^−1^ (Figure 2E), while a slow, but constant decline in Φ_PSII_ was observed by 14% between 4 and 29 h after biotransformation start (Figure 2H). The sudden decrease in growth rate to 0.048 ± 0.007 h^−1^ 48 h after the biotransformation start, together with a strong decrease in Φ_PSII_ to 0.131 ± 0.037 at 52.6 h, indicates that cell metabolism became heavily affected upon continuous withdrawal of reduction equivalents for biotransformation. Like in scenario I, biotransformation performance concomitantly was reduced, leading to substrate accumulation (Figure 3B). Interestingly, growth recovered as soon as biotransformation stalled (from 69 h on). In conclusion, although arithmetically possible, the reduced metabolic electron demand with NH_4_^+^ as N‐source did not sustain continuous oxygenase catalysis. Possibly, combined effects of NH_4_^+^, Ni^2+^, and the reaction itself posed too much burden to the cellular metabolism in this scenario.

### 3.4 Low‐Carbon Conditions Enable Long‐Term Biotransformation at Reduced Conversion Rate

In a third scenario, CO_2_ supply was reduced to a fifth of the previous concentration (0.12% (v/v) in IN‐gas), while NO_3_^−^ was used as N‐source to avoid possible NH_4_^+^‐related stress. This ensured a reduction of the metabolic electron demand by sink limitation, as the CO_2_ fixation rate was thereby reduced at the same light intensity of 150 μmol_photons_ m^−2^ s^−1^ potentially enabling the coverage of biotransformation‐related electron demands (see above). This is illustrated by a low μ of 0.034 ± 0.002 h^−1^ under non‐induced control conditions (Figure 2C). Both *Q*_phar_ (41.7 ± 2.3 mmol e^−^ g_CDW_^−1^ h^−1^, Figure 2F) and Φ_PSII_ (0.302 ± 0.038, Figure 2I) were relatively low, showing that photosynthesis did not operate at its full capacity as expected for sink‐limited conditions. Nevertheless, Ni^2+^‐dependent induction of heterologous gene expression caused a slight decrease in growth rate (15%), while both *Q*_phar_ and Φ_PSII_ increased by 14% and 17%, respectively.

To account for the assumed lower electron fluxes under sink‐limited conditions, the C‐one feed rate was reduced to a rate corresponding to 8.4 U g_CDW_^−1^, still corresponding to a higher percentage (21%) of the r_C_ in induced cells compared to the above‐described source‐limited conditions. As C‐one started to accumulate, the feed rate was reduced to a rate corresponding to a specific activity of 5 U g_CDW_^−1^, 12.5% of the r_C_, after 16 h. Within the first 24 h of biotransformation, μ remained constant between 0.031 and 0.034 h^−1^ (Figure 2C), while *Q*_phar_ and Φ_PSII_ remained at a comparably high level (Figure 2F,I). Interestingly, a strong decline of μ after 48 h was not observed with a constant μ of 0.021–0.022 h^−1^ between 72 and 120 h. This and the constant *Q*_phar_ (between 40.6 and 46.8 mmol e^−^ g_CDW_^−1^ h^−1^) and Φ_PSII_ (between 0.30 and 0.34) in the same time range indicated a higher metabolic stability upon sink limitation. However, the specific activity nevertheless slowly decreased (~2.5 U g_CDW_^−1^ after 112 h of biotransformation) leading to C‐one accumulation (Figure 3C), which finally also affected *μ* and Φ_PSII_ and thus continuous cultivation stability.

### 3.5 Electron Fluxes in *Synechocystis* BVMO:Lactonase

In a phototrophic cell, a supply of reductant and energy is required for nutrient assimilation, cell growth, and maintenance. The BVMO reaction consumes additional reductants, which disturbs the established source/sink balance. The goal of this study was to quantify electron fluxes in oxygenase‐active cyanobacteria to identify main pitfalls and potentials for electron‐consuming biotransformations in the long term.

In order to quantify the electron fluxes under different conditions, the electron demands for growth and biotransformation were calculated as follows: based on nutrient supply, elemental composition of the biomass, and growth rate, the assimilation costs were translated into electron fluxes toward carbon fixation (r_C_) and nitrogen assimilation (r_N_) as the main electron sinks. With a sulfur mass fraction of < 0.5% (Touloupakis et al. 2016), the respective assimilation costs are negligible as it is the case for other nutrients. The electron flux into product formation (r_BT_) can be quantified based on the reaction stoichiometry of 2 mol e^−^ per mol product. Since the alternative electron transfer, which comprises Mehler‐like reactions and other dissipative mechanisms, cannot be directly quantified, the maximum electron flux through PSII (r_F_) was assessed via PAM‐fluorometry as previously described (Grund et al. 2019), and r_NEF_ was defined as the difference between r_F_ and the sum of all assimilation‐ and biotransformation‐related electron fluxes. The calculated electron fluxes for the three different sink scenarios discussed above are depicted in Figure 4.

**FIGURE 4:**
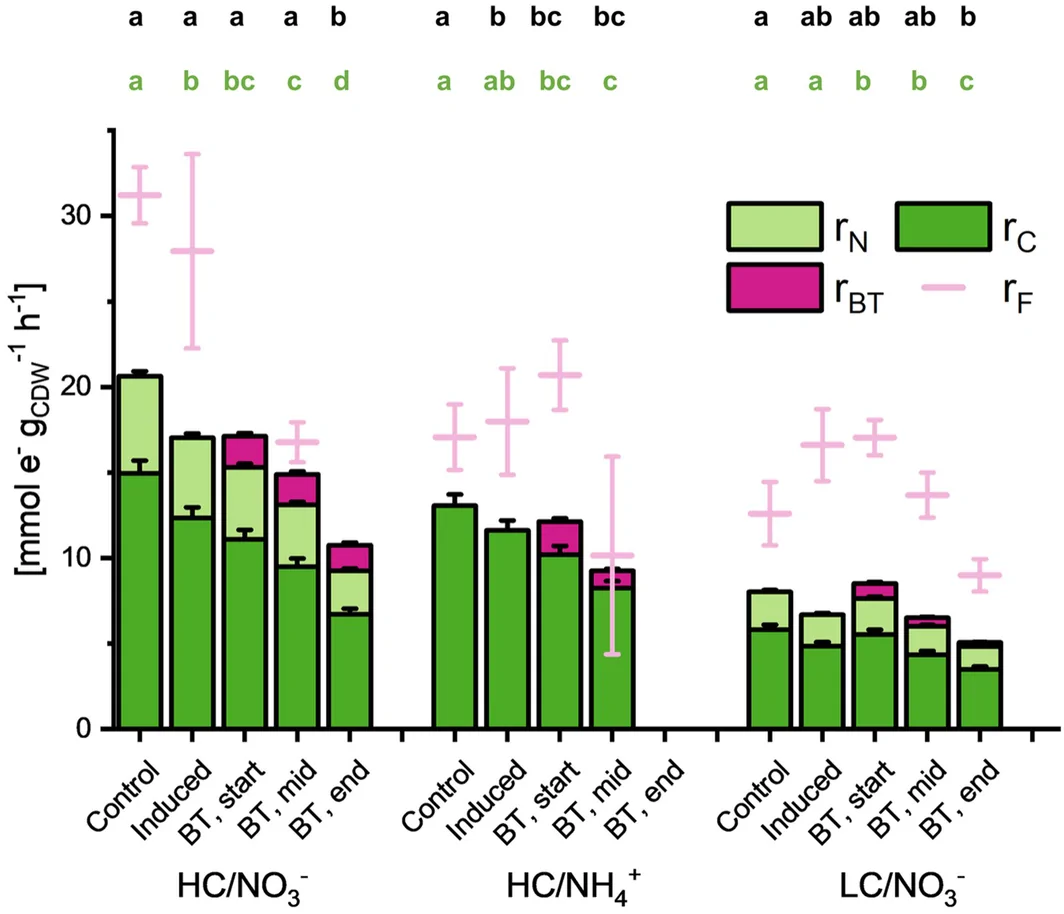
Electron balancing under HC/NO_3_^−^, HC/NH_4_^+^, and LC/NO_3_^−^ conditions. For non‐induced (“Control”) and induced conditions (“Induced”), steady state data for electron fluxes are shown. For biotransformation conditions (BT), three phases (start = 0–25 h; mid = 40–52 h; end = 72–120 h after start of substrate feed) are depicted. r_F_, maximum electron flux through PSII, based on fluorometric measurements; r_C_ and r_N_, electron fluxes toward carbon and nitrate assimilation, respectively; r_BT_, electron flux into product formation. Means and standard deviations from ≥ 3 technical replicates are given. Statistical differences among the conditions were evaluated separately for the parameters μ (green letters) and Q_phar_ (black letters) using one‐way analysis of variance (ANOVA), followed by Turkey's multiple comparison test. Results are presented as compact letter displays, where groups sharing the same letter are not significantly different (*p* < 0.05). See experimental section for details. HC—high carbon, LC—low carbon.

For both control conditions (non‐induced and induced), steady state data are presented, while the biotransformation conditions are presented by all recorded data from 0 to 50 h of biotransformation (see also Table 1). The overall highest r_F_ was observed in the growth control under HC/NO_3_^−^‐conditions. Upon Ni^2+^‐induction, r_F_ decreased by 7%, while growth rate reduction was more pronounced (17%, Figure 2A). As control experiments with the wildtype strain show (Table 1, Figure S3), Ni^2+^‐effects alone do not explain the growth effects. Upon biotransformation, a strong decrease in r_F_ was observed, accompanied by an intensified competition for electrons. Consequently, the high growth rate decreased, correlating with the reduced electron flux into C‐ and N‐assimilation.

The second scenario with NH_4_^+^ instead of NO_3_^−^ as N‐source relieved the reductant demand for N‐assimilation. This maintained a comparably high growth rate (Figure 2B) with only 55% of the r_F_ observed in sink scenario I (Figure 4). Upon induction, r_F_ increased by 4%, whereas μ was reduced by 22%, resulting in an increase of r_NEF_ possibly due to the expression burden including oxidative stress, which may be caused by uncoupled incomplete O_2_ reduction by the BVMO. Upon biotransformation initiation, the r_F_ was kept constant only for a short time period before Φ_PSII_ decreased dramatically (see Figure 2E), resulting in a drastic decrease in r_F_ and consequently in *μ*.

In the LC/NO_3_^−^‐scenario, the lowest r_F_ was observed (Figure 4), which correlates with a low Φ_PSII_ (Figure 2I). The electron partitioning, however, scales well with the HC/NO_3_^−^‐conditions (Figure 4). In contrast to this scenario, cells were able to increase r_F_ (+34%) upon Ni^2+^‐induction, going along with only a slight decrease of μ by 14%. Only under LC/NO_3_^−^‐conditions, r_F_ could be kept high in the first 50 h of biotransformation (−20% compared to induced control conditions). However, a sink effect of the biotransformation (relief of sink limitation) was not observed: neither r_F_ nor μ were stably increased during the biotransformation. In fact, from 48 h onwards, r_F_ decreased slowly together with μ. Finally, the specific activity also decreased, leading to a C‐one accumulation and consequently to a decrease in both μ and r_F_.

## 4 Discussion

### 4.1 Physiological Responses to an Extra Electron Sink Depend on Source–Sink Conditions

Ni^2+^‐induction reduced growth under all tested conditions, which is likely attributable to the metabolic burden of heterologous gene expression, as control experiments with the wildtype strain showed no reduction in growth (Figure S3). Previous work with a similar construct estimated expression at ~1.1% ± 0.2% of total soluble protein (Tüllinghoff et al. 2022), consistent with the modest growth reduction observed under HC/NO_3_^−^ (sink‐replete) and LC/NO_3_^−^ (sink‐limited) conditions. However, the pronounced growth inhibition in *Synechocystis* BVMO:Lactonase under HC/NH_4_^+^ conditions cannot be explained by expression burden alone. Combined effects of expression cost and NH_4_^+^ toxicity were likely detrimental, doubling r_NEF_ and imposing high energetic demands. NH_4_^+^ is known to enhance PSII photodamage, potentially by destabilizing the Mn cluster of the O_2_‐evolving complex (Drath et al. 2008; Dai et al. 2014). This may restrict electron flux through the photosynthetic electron transport chain (PETC) and thereby limit ATP and NADPH generation. Simultaneously, detoxification processes such as Ni^2+^ export and PSII repair (Aro et al. 1993) would further increase energy requirements, explaining the observed rise in r_NEF_.

In none of the tested scenarios, the two‐step biotransformation reaction, used as a proxy for electron withdrawal, could be stabilized in metabolic steady states:

1. Sink‐replete (HC/NO_3_^−^, scenario I): Under light limitation, electron supply was likely maximized to meet growth‐associated demand. Biotransformation thus competed with essential metabolic sinks, forcing redox and energy rebalancing. This intense competition reduced *μ* after initiation upon induction and biotransformation. The inability to maintain the lowered μ during biotransformation, involving concurrent declines in Φ_PSII_ and *Q*_phar_, remains unexplained.
2. NH_4_^+^ as N‐source (HC/NH_4_^+^, scenario II): Compared to NO_3_^−^ utilization as N‐source, NH_4_^+^ assimilation requires fewer electrons, but imposes strong stress upon Ni^2+^ induction, likely due to detoxification and PSII repair costs. The metabolism failed to accommodate the additional sink. Initiation of biotransformation in this fragile state triggered rapid loss of growth and Φ_PSII_, suggesting the irreversible breakdown of metabolism.
3. Sink‐limited (LC/NO_3_^−^, scenario III): Lower substrate supply reduced biotransformation flux, explaining the relative stability of *μ*, *Q*_phar_, and Φ_PSII_ for ~100 h. However, the anticipated increase in PETC flux upon partial biotransformation‐based sink relief did not occur. Substrate accumulation even at low substrate feed rates suggests an upper limit to biotransformation, potentially due to down‐regulation of metabolic turnover under sink limitations.

### 4.2 Stability of Oxygenase Biocatalysis Fueled by Phototrophic Metabolism

In all tested scenarios, biotransformation was driven by electrons derived from the light reaction, particularly during the first 24 h. Throughout the experiments, electrons diverted to the heterologous sink reduced their availability for assimilation, as reflected in growth impairment across conditions. This was most evident under source limitation (scenario I), when competition between growth and biotransformation was strongest. Despite this, BVMO was supplied with electrons, but operating below *V*_max_ [1.2 ± 0.1 U mg^−1^] (Bretschneider et al. 2021) and below maximal whole‐cell activities up to 63 U gCDW^−1^ (Tüllinghoff et al. 2023). Enzyme kinetics indicate a higher NADPH‐affinity of the CBB enzyme GAPDH (K_M_ = 40 ± 3 and 62 ± 4.5 μM for *Thermosynechococcus elongatus* (Mcfarlane et al. 2019) and *Synechococcus* PCC 7942 (Tamoi et al. 1996), respectively) than of BVMO (K_M_ = 372 ± 17 μM; Bretschneider et al. 2021). However, both enzymes may be saturated at estimated intracellular NADPH concentrations of 0.3–1 mM (Tanaka et al. 2021). Thus, carbon fixation was unlikely limited by the availability of NADPH, ATP, that is adaptable via cyclic electron flux, or CO_2_, which was provided in excess. Instead, CBB cycle regulation or carbon allocation may have a constrained fixation. At later stages, when growth and biotransformation both declined, a metabolic imbalance—likely an ATP/NADPH imbalance—appears to have caused NADPH limitation.

BVMO instability at low NADPH/NADP^+^ ratios may further compromise activity. In vitro, CHMO stability was shown to depend on NADPH supplementation (Goncalves et al. 2017). *Synechocystis* maintains a NADPH/NADP^+^ ratio of around 2 under light conditions in vivo (Kauny and Setif 2014; Tanaka et al. 2021), which is sensitive to perturbations by heterologous sinks. Thus, electron withdrawal likely decreased this ratio, possibly destabilizing BVMO. High light may promote NADPH excess and thereby enzyme stability, but in our setup induced biofilm formation prohibiting continuous cultivation.

A key regulatory parameter of photosynthetic metabolism is the ATP/NADPH ratio (Nogales et al. 2012; Kugler and Stensjö 2023). Linear PETC flux generates ATP and NADPH at a ratio of ~1.28 (Kramer and Evans 2011), lower than the 1.5 required by the CBB cycle. Cyclic and pseudo cyclic fluxes adjust this ratio by increasing the ATP share (Allen 2003; Nogales et al. 2012). Heterologous sinks increasing the electron demand by 10%, like in the experiments of this study, would decrease the required ratio to ~1.35, which, although being still above the ratio expected from linear PETC flux, may be below the balancing capacity of phototrophic metabolism (Santos‐Merino et al. 2021). Consistent with this, moderate electron withdrawal in our previous BVMO cascade study improved stability, whereas high withdrawal induced metabolic stalling, likely due to ATP excess (Tüllinghoff et al. 2023). Photorespiration could alleviate ATP excess, but Rubisco oxygenation—and thus photorespiration—was minimal under the low O_2_ conditions used here. Indeed, low r_NEF_ under HC/NO_3_^−^ suggests reduced cyclic flux, consistent with prior reports that strong heterologous sinks suppress cyclic electron transfer (Hubacek et al. 2024).

By contrast, under HC/NH_4_^+^ conditions, r_NEF_ increased after induction, reflecting elevated energy demand. Then, declining biotransformation correlated with a reduced PETC flux, likely due to NH_4_^+^‐induced PSII photodamage (Drath et al. 2008; Dai et al. 2014). Neither linear nor cyclic electron transfer could support biotransformation, detoxification, and PSII repair, culminating in metabolism breakdown. Under LC/NO_3_^−^‐conditions, reduced substrate supply enabled the longest stabilization. Future studies should quantify intracellular ATP and NADPH pools using LC–MS (Lu et al. 2018; Tanaka et al. 2021) or in situ sensors (Tao et al. 2017; Tantama et al. 2013) to directly assess respective (im)balances.

Knockout of competing electron sinks, such as flavodiiron proteins (Flv), has been proposed as a strategy to stabilize electron allocation toward heterologous biotransformations. For example, deletion of Flv3 (thereby disrupting the Flv1/3 complex) decreases the ATP/NADPH ratio (Thiel et al. 2019), enhances sucrose synthesis under low light conditions (Thiel et al. 2019), and doubles the activity of an NADPH‐dependent ene‐reductase (Assil‐Companioni et al. 2020). However, Flv1/3 also functions as an electron valve and supports ATP synthesis during dark–light transitions. Consequently, its deletion compromises robustness under fluctuating light conditions (Nikkanen et al. 2021). Further, recent work suggests that sufficiently strong heterologous sinks can already outcompete alternative acceptors without requiring their genetic removal (Hubacek et al. 2024). Together, these findings question whether downregulation of endogenous electron sinks is a generally effective strategy for stabilizing biotransformations, particularly given the associated loss of cellular robustness.

### 4.3 Balancing of Phototrophic Metabolism Between Energy Source and Metabolic Sinks

As outlined above, light‐driven metabolism requires continuous balancing between energy source (light) and metabolic sinks (assimilation, maintenance, and biotransformation). Energy and electron supply primarily depend on light availability. Up to a threshold intensity (*E*_k_), growth increases proportionally with irradiance but saturates at higher intensities, particularly under low CO_2_, reflecting sink limitation (Figure 2; Zavrel et al. 2019). In this study, light intensity was held constant at levels supporting efficient planktonic growth, while environmental sink conditions—carbon source availability, nitrogen source type, expression burden, and biotransformation load—were systematically varied. These factors influenced not only growth but also electron partitioning, most prominently the relative non‐assimilatory electron flux (r_NEF_).

In *Synechocystis* BVMO:Lactonase, the r_NEF_ fraction accounted for ~35% under non‐induced control conditions with NO_3_^−^ as nitrogen source, but only for ~20% with NH_4_^+^. Ni^2+^‐induced heterologous expression increased r_NEF_ under all conditions (including wildtype control, see Figure S3), reaching up to 60% under LC/NO_3_^−^, indicating substantial expression burden and increased ATP demand. These results confirm the inherent flexibility of photosynthetic electron allocation in response to altered energetic and redox demands. Unexpectedly, while biotransformation reduced r_NEF_ as anticipated, it also lowered total electron flux (r_F_), impairing both growth and catalytic activity.

Carbon fixation efficiency, expressed as the quantum requirement for carbon fixation *Q*_C_ [mol_quanta_ mol_C_^−1^], serves as an indicator of source–sink balance (Grund et al. 2022). The lowest *Q*_C_ (28.3 ± 2.9 mol_quanta_ mol_C_^−1^) under HC/NH_4_^+^ conditions reflected the most balanced state, slightly superior to HC/NO_3_^−^ (31.0 ± 2.9), where sink demands were higher due to costs for N assimilation (Table 1). Initiation of biotransformation increased Q_C_ across all conditions, reflecting a reduced balance. Under sink‐replete HC/NO_3_^−^ conditions, introduction of an additional electron sink nearly increased Q_C_ by ~50%, concomitant with decreased r_F_—likely resulting from an unfavorable ATP/NADPH ratio. In scenario II (HC/NH_4_^+^), NH_4_^+^‐induced photodamage further reduced r_F_, again increasing Q_C_ by ~50%. Under sink‐limited conditions, the already high Q_C_ (57.5 ± 6.1 mol_quanta_ mol_C_^−1^) rose by an additional 25% upon biotransformation, indicating that electron withdrawal did not relieve sink limitation but rather suppressed r_F_, likely due to an ATP/NADPH imbalance.

Notably, the lowest *Q*_C_ during biotransformation coincided with the highest quantum efficiency for product formation QE_6‐HA_ (Table 1), both observed under HC/NO_3_^−^ conditions. Thus, maximal biotransformation capacity occurred under carbon‐replete conditions, arguing against direct competition for reductants between carbon/nitrogen assimilation and the heterologous reaction. Q_C_ therefore represents a useful indicator for evaluating process efficiency in systems utilizing photosynthetically derived reducing power. Consistent with previous work, conditions that support rapid growth also favor efficient production (Tüllinghoff et al. 2023). In contrast to the previously reported fed‐batch process, in which biotransformation enhanced overall quantum utilization and exploited unused photosynthetic capacity, the continuous, light‐limited setup employed here did not evoke such an effect. This signifies that light limitation constitutes an unfavorable regime: rather than stimulating additional electron flux, catalysis proceeded at the expense of electrons otherwise directed toward carbon and nitrogen assimilation.

## 5 Conclusions

By balancing photosynthesis‐derived electrons, this study enabled access to physiological parameters like growth rate, electron fluxes, and electron partitioning toward growth during heterologous biotransformation of cyclohexanone to 6‐hydroxyhexanoic acid in different source/sink scenarios. Despite being desirable for many envisioned photo‐biotechnological processes, it turned out not to be trivial to sustainably tap electrons from PS I at high rates for the heterologous biotransformation. Sink‐replete conditions (HC/NO_3_^−^) were found to be ideal to enable high electron withdrawal rates. In our setup, however, long‐term stability was impaired. Stability of the light‐driven biotransformation could be extended up to several days under sink‐limited (LC/NO_3_^−^) conditions, albeit at lower conversion rates. Overall, we could not find conditions to continuously maintain the biotransformation during continuous cultivation. While there is evidence for an electron‐sink effect of a heterologous consumer, the interdependency of electron‐demanding biotransformations and photosynthetic growth metabolism as well as respective regulations were found to be of key importance for stable processing and require further investigation. This study shows the importance of carefully considering source/sink balances for continuous light‐driven production in cyanobacteria. In particular, respective long‐term effects appear to play a crucial role, of which the characterization and overcoming will be imperative for possible future photo‐biotechnological applications.

## Author Contributions

Adrian Tüllinghoff, Bruno Bühler, and Jörg Toepel were involved in the conceptualization and design of the study. Adrian Tüllinghoff and Lieselotte Van Looveren conducted experimental work and data collection. Adrian Tüllinghoff curated and analyzed the data; Bruno Bühler and Jörg Toepel supported in terms of data validation. Adrian Tüllinghoff wrote the original draft of the manuscript. Bruno Bühler and Jörg Toepel contributed in terms of reviewing and editing the manuscript. All authors were involved in final editing and approved the submitted version.

## Funding

This work was supported by the European Union and the State of Saxony via the Just Transition Fund (JTF) InfraProNet 2021–2027, grant no. 10702818.

## Disclosure

During the preparation of this manuscript, the authors used generative artificial intelligence (AI) tools to assist with improving the language, clarity, and structure of the manuscript. AI tools were used for editorial support, including suggesting improvements to the wording and organization of the text. No AI tool was used to generate scientific data, results, figures, analyses, or conclusions. All AI‐assisted content was critically reviewed, verified, and edited by the authors. The authors take full responsibility for the accuracy, originality, integrity, and scientific validity of the final manuscript. The use of AI was conducted in accordance with the applicable policies of the AI tools, with appropriate consideration of attribution and copyright requirements. The authors also assessed the AI‐assisted content for potential inaccuracies, misleading information, and unintended bias.

## Conflicts of Interest

The authors declare no conflicts of interest.

## Supporting information


**Figure S1:** Dependency of the growth rate on light intensity. *Synechocystis* cultures were grown in flat panel bioreactors under steady state conditions as described in the Materials and Methods section. For every light intensity, a new steady state was established, including at least three reactor volume exchanges at constant cell density.
**Figure S2:** Specific absorption shifts upon Ni2+‐induction under HC/NO3− (pink), HC/NH4+ (green), and LC/NO3− (magenta) conditions. Absorption spectra were recorded from 400 to 700 nm at 1 nm intervals using a GENESYS 150 UV/Vis spectrophotometer (Thermo Fisher Scientific, Waltham, MA, USA). Spectra were normalized to biomass concentration. The plotted ΔA(λ) represents the change in absorbance following Ni2+ induction relative to the pre‐induction spectrum. Under HC/NH4+ conditions, the observed spectral changes indicate a decrease in chlorophyll a (440 and 680 nm) and phycobiliproteins (625 nm).
**Figure S3:** Electron balancing with *Synechocystis* wildtype without (−Ni2+) and with (+Ni2+) 5 μM NiSO4. Steady state data for electron fluxes under HC/NO3− conditions are depicted. rNEF, non‐sink‐related electron flux; rC and rN, electron flux for carbon and nitrate assimilation, respectively. Data are depicted as mean values and standard deviations from at least three replicates. See experimental section for details.
**Figure S4:** Cyclohexanone (C‐one) toxicity on *Synechocystis* wildtype cells. Cultures were grown in 100 mL Multicultivators (Photon System Instruments, Drásov, Czech Republic) under continuous illumination at 50 μmolphotons m−2 s−1 with 1% CO2 and a total aeration rate of 1 vvm. C‐one was added 24 h after inoculation at the indicated transformations. The relative specific growth rate (μ) was calculated from the exponential growth phase between 27 and 40 h normalized to the growth rate prior to C‐one addition. Data are presented as mean values and standard deviations from at least three replicates.

## Data Availability

The data that support the findings of this study are available from the corresponding author upon reasonable request.
