# Supplementary material for: From Photons to Product – A Quantitative Study on Electron Fluxes in Synechocystis sp. PCC 6803 Applied for Oxygenase Catalysis
Source: Physiol Plant. 2026 Oct 13;178(5):e71126. doi: 10.1111/ppl.71126 (PMC13659377; doi:10.1111/ppl.71126)
Supplement: Supplementary file 1 [file PPL-178-e71126-s001.docx]

## Supplementary information for

**From photons to product – a quantitative study on electron fluxes in *Synechocystis* sp. PCC 6803 applied for oxygenase catalysis**

Adrian Tüllinghoff^1^, Lieselotte Van Looveren^1^, Jörg Toepel^1,*^, and Bruno Bühler^1^

^1^Department of Microbial Biotechnology, Helmholtz-Centre for Environmental Research – UFZ Leipzig, Germany

**List of abbreviations**

| (MC-)PAM | (multi-color) pulse amplitude modulation |
| --- | --- |
| 6-HA | 6-hydroxy hexanoic acid |
| BVMO | Baeyer-Villiger-monooxygenase |
| C | carbon |
| CBB | Calvin-Benson-Bassham |
| CDW | cell dry weight |
| Chl-*a* | Chlorophyll *a* |
| C-ol | cyclohexanol |
| C-one | cyclohexanone |
| ε-Cl | ε-caprolactone |
| E_k_ | Light saturation point |
| FP | Flat-panel |
| GC | Gas chromatography |
| HC | high carbon |
| HEPES | 4-(2-hydroxyethyl)-1-piperazinethanesulfonic acid |
| HPLC | High pressure liquid chromatography |
| LC | low carbon |
| N | nitrogen |
| PETC | photosynthetic electron transport chain |
| PSII | Photosystem II |
| Q_c_ | quantum requirement for carbon fixation |
| QE | quantum efficiency |
| Qphar | quantum absorption |
| r_NEF_ | Non-sink accounted electron flux |
| r_BT_ | electron flux into product formation |
| r_C_ | electron flux into carbon fixation |
| r_F_ | overall photosynthetic electron transfer at PSII |
| r_N_ | electron flux into nitrogen assimilation |
| Φ_PSII_ | effective quantum yield at PSII |

Figure S1: **Dependency of the growth rate on light intensity**. *Synechocystis* cultures were grown in flat panel bioreactors under steady state conditions as described in the Materials and Methods section. For every light intensity, a new steady state was established, including at least three reactor volume exchanges at constant cell density.

Figure S2: **Specific absorption shifts upon Ni^2+^-induction under HC/NO_3_^-^ (pink), HC/NH_4_^+^ (green), and LC/NO_3_^-^ (magenta) conditions.** Absorption spectra were recorded from 400 to 700 nm at 1 nm intervals using a GENESYS 150 UV/Vis spectrophotometer (Thermo Fisher Scientific, Waltham, MA, USA). Spectra were normalized to biomass concentration. The plotted ΔA(λ) represents the change in absorbance following Ni²⁺ induction relative to the pre-induction spectrum. Under HC/NH₄⁺ conditions, the observed spectral changes indicate a decrease in chlorophyll *a* (440 and 680 nm) and phycobiliproteins (625 nm).

Figure S3: **Electron balancing with *Synechocystis* wildtype without (- Ni^2+^) and with (+ Ni^2+^) 5 µM NiSO_4_.** Steady state data for electron fluxes under HC/NO_3_^-^ conditions are depicted. r_NEF_, non-sink-related electron flux; r_C_ and r_N_, electron flux for carbon and nitrate assimilation, respectively. Data are depicted as mean values and standard deviations from at least three replicates. See experimental section for details.

Figure S4: **Cyclohexanone (C-one) toxicity on *Synechocystis* wildtype cells.** Cultures were grown in 100 mL Multicultivators (Photon System Instruments, Drásov, Czech Republic) under continuous illumination at 50 µmol_photons_ m^-2^ s^-1^ with 1% CO_2_ and a total aeration rate of 1 vvm. C-one was added 24h after inoculation at the indicated transformations. The relative specific growth rate (µ) was calculated from the exponential growth phase between 27 to 40h normalized to the growth rate prior to C-one addition. Data are presented as mean values and standard deviations from at least three replicates.
